# Safety and Tolerability of Sodium Thiosulfate in Patients with an Acute Coronary Syndrome Undergoing Coronary Angiography: A Dose-Escalation Safety Pilot Study (SAFE-ACS)

**DOI:** 10.1155/2020/6014915

**Published:** 2020-09-24

**Authors:** Marie-Sophie L. Y. de Koning, Solmaz Assa, Carlijn G. Maagdenberg, Dirk J. van Veldhuisen, Andreas Pasch, Harry van Goor, Erik Lipsic, Pim van der Harst

**Affiliations:** ^1^University of Groningen, University Medical Center Groningen, Department of Cardiology, Groningen, 9713 GZ, Netherlands; ^2^Institute for Physiology and Pathophysiology, Johannes Kepler University, Linz 4040, Austria; ^3^Lindenhofhospital, Department of Nephrology, Bern 3011, Switzerland; ^4^Nierenpraxis Bern, Bern 3011, Switzerland; ^5^University of Groningen, University Medical Center Groningen, Department of Pathology and Medical Biology, Groningen, 9713 GZ, Netherlands; ^6^Department of Cardiology, University Medical Center Utrecht, Utrecht, 3584 CX, Netherlands

## Abstract

**Background:**

In animal studies, hydrogen sulfide (H_2_S) has been shown to protect the heart from ischemia-reperfusion injury. This study evaluates the safety and tolerability of the H_2_S donor sodium thiosulfate (STS) in patients with acute coronary syndrome (ACS).

**Methods:**

Eighteen patients, undergoing coronary angiography for ACS, received STS intravenously immediately after arrival at the catheterization laboratory according to a “3 + 3 dose-escalation design” with fixed dosing endpoint (0, 2.5, 5, 10, 12.5, and 15 grams). This first dose STS was combined with verapamil and nitroglycerin required for transradial procedures. A second dose STS was administered 6 hours later. Primary endpoint was dose-limiting toxicity, defined as significant hemodynamic instability or death up to 24 hours or before discharge from the coronary care unit. Secondary outcomes included the occurrence of anaphylaxis, nausea, vomiting, and systolic blood pressure (SBP) course.

**Results:**

Sixteen patients received two dosages of STS and two patients one dosage. None of the patients reached the primary endpoint, nor experienced a serious adverse event. We observed a clinically well-tolerated decline in SBP 1 hour after administration of the first STS dose and concomitant verapamil/nitroglycerin. SBP for all patients together reduced 16.8 (8.1–25.5) mmHg (*P* = 0.0008). No significant decline in SBP occurred after the second dose. Mild nausea was observed in one patient.

**Conclusion:**

This is the first report on sodium thiosulfate administration in patients with acute coronary syndromes. Our data suggest that sodium thiosulfate was well tolerated in this setting. The potential benefit of this intervention has to be examined in larger studies.

## 1. Introduction

Timely and effective reperfusion by primary percutaneous coronary intervention (PPCI) is currently the most effective treatment of ST-segment elevation myocardial infarction (STEMI). However, reperfusion not only saves the majority of the ischemic cells but also has a downside, which might paradoxically lead to additional myocardial injury and cardiomyocyte death [1, 2]. Therefore, new treatments against ischemia-reperfusion injury may be effective to further reduce myocardial infarct size and prevent the onset of heart failure [3, 4].

Administration of hydrogen sulfide (H_2_S), an endogenous gaseous signaling molecule, has been shown to protect the heart from ischemia-reperfusion injury in various experimental models. It reduces infarct size and apoptosis and attenuates the loss of cardiac function. Inhibition of leukocyte endothelial cell interactions, neutralization of reactive oxygen species (ROS), and the reduction of apoptotic signaling were all suggested as mechanisms underlying the cardioprotective effect of H_2_S in this setting [5–15].

H_2_S is synthesized endogenously by enzymatic and nonenzymatic pathways. One of the intermediate endogenous metabolites of H_2_S in the nonenzymatic pathway is thiosulfate. Especially under hypoxic conditions, thiosulfate is also able to produce H_2_S [16]. Furthermore, additional anti-inflammatory and antioxidant effects of thiosulfate are related to its reaction with mitochondrial thiosulfate sulfurtransferase [17]. As a result of this interaction, sulfur transfers into gluthation and thioredoxin, thereby promoting thiol-dependent antioxidative mechanisms, resulting in additional ROS scavenging. Thiosulfate can also be exogenously administered as sodium thiosulfate (STS), and next to its antioxidant properties, also H_2_S-related mitochondrial preservation and reduced apoptosis are experimentally proven cardioprotective mechanisms [18–22].

Data in humans show that sodium thiosulfate can be administered safely and effectively for non-cardiac indications: STS is used in humans since 1933 for the treatment of cyanide intoxication and since the 1980s for treatment of vascular calcifications in end-stage renal disease (calciphylaxis) [23, 24]. It is also used to prevent ototoxicity of cisplatin treatment [25, 26]. The mechanisms of action in these diseases are thought to be based on potential calcium chelating and antioxidant properties of STS. In most cases, intravenous STS was used in different doses from 10 to 25 grams per day. Most side effects were mild and manageable and comprised nausea and vomiting, hypotension, and hypernatremia. In 8%–15% of hemodialysis patients, metabolic acidosis occurred [27].

Preclinical and clinical data on sodium thiosulfate are encouraging to investigate the efficacy of sodium thiosulfate on myocardial infarct size. However, STS administration has not yet been tested in the clinical setting of an acute coronary syndrome (ACS), and data regarding the safety and tolerability of STS in patients with ACS undergoing coronary angiography (CAG) via a transradial route are lacking; especially the effects on blood pressure of concomitant administration of STS and vasodilating drugs during CAG are of interest. Moreover, interactions of STS with other cardiac drugs are unknown.

The SAFE-ACS trial is a phase 1, open-label, dose-escalation study to test the hypothesis that STS, on top of standard medical treatment, can be safely administered and is well tolerated in patients presenting with an ACS, undergoing CAG via transradial route with coadministration of verapamil and nitroglycerin. Safety and tolerability were evaluated by assessing dose-limiting toxicity (DLT) and the maximum tolerable dose (MTD) using a “3 + 3 design” with a fixed dosing endpoint.

## 2. Methods

### 2.1. Study Population

Patients, ≥18 years old, presenting with acute coronary syndrome (ACS) at the University Medical Center Groningen (UMCG) between October 2017 and March 2018 during office hours were screened for enrollment. Inclusion criteria were the diagnosis of ACS defined by chest pain suggestive for myocardial ischemia for at least 30 minutes, with time from onset of the symptoms less than 24 hours before hospital admission, with or without an electrocardiogram (ECG) recording with ST-segment elevation of more than 0.1 millivolt in 2 or more contiguous leads. Furthermore, patients had to undergo CAG via a transradial approach. Exclusion criteria were the presence of a cardiomyopathy or impaired LV-ejection fraction <35%, systolic blood pressure (SBP) <100 mmHg or >180 mmHg at presentation, pregnancy/breastfeeding, a recent (<1 year) malignancy treated with chemo- and/or radiotherapy, and a condition with a life expectancy of less than 1 year or any condition which does not allow the patient to successfully participate in the study.

This study (http://www.clinicaltrials.gov, NCT03017963) was approved by the local ethics committee (Groningen, the Netherlands), and all patients provided written informed consent.

### 2.2. Study Procedures

At admission to the hospital, baseline laboratory and vital parameters were assessed. Prior to coronary angiography, at the catheterization laboratory, the first dose of STS was administered intravenously in 15 minutes. Six dose cohorts were created. Each cohort included three patients receiving, respectively, 0 gram, 2.5 grams, 5 grams, 10 grams, 12.5 grams, and 15 grams of STS dissolved in 250 ml sodium chloride 0.9%. Hemodynamic parameters and occurrence of side effects were cautiously monitored. CAG was performed via a transradial approach using standard techniques, including administration of 2.5 mg verapamil and 200 mcg nitroglycerin. Number of diseased vessels was defined during CAG as ≥70% reduction by plaque or stenosis in the internal diameter of the right coronary artery, left anterior descending or left circumflex coronary artery or ≥50% reduction in the internal diameter of the left main coronary artery. In the absence of dose-limiting toxicity, a second dose of STS was administered 6 hours after the first dose at the coronary care unit (CCU). The duration of the second infusion was 30 minutes. Patients were followed up to 24 hours after treatment with study medication or until discharge from CCU. A schematic overview of the study design is presented in Figure S1. All study procedures took place at the UMCG. STS was produced by A15 Pharmacy (Gorinchem, the Netherlands).

### 2.3. Primary and Secondary Endpoints

This study was designed to assess safety and MTD. Primary endpoint was the development of DLT during or after STS administration up to 24 hours or discharge from the CCU. DLT was defined as all-cause mortality or hemodynamic instability of clinical significance. The latter was defined according to the established criteria for cardiogenic shock: (i) SBP <90 mmHg for >30 min and/or vasopressors required to achieve a blood pressure ≥90 mmHg; (ii) pulmonary congestion or elevated left ventricular filling pressures; and (iii) signs of impaired organ perfusion with at least one of the following criteria: (a) altered mental status; (b) cold and clammy skin; (c) oliguria (urine output <30 ml/h); and (d) increased serum-lactate >2.0 mmol/L [28].

Secondary outcomes included the occurrence of anaphylaxis, nausea, vomiting, and the prescription of metoclopramide. An anaphylactic reaction was defined according to the World Allergy Organization guidelines for the assessment and management of anaphylaxis [29]. Nausea and vomiting was monitored during and after medication infusion with a 4-point Likert scale for severity (none, mild, moderate, and severe). Furthermore, blood pressures were evaluated before and once every hour during the first 3 hours after each dose of STS. Hypotension was especially important to monitor because this patient population is prone to develop hemodynamic instability, due to disease and performed interventions, and possible interactions of STS with the vasodilator cocktail, required for CAG, are unknown.

### 2.4. Sample Size Considerations

Sample size is based on the classic 3 + 3 design with fixed dosing endpoint of 15 grams STS. Three patients were enrolled in every dosing cohort. In case that one out of three patients would develop DLT at a specific dose, an additional three subjects would be enrolled into the same dose cohort (maximum six per cohort). If none of these patients experienced DLT, enrollment to the next cohort was allowed. When more than one out of six patients developed DLT, the trial would be terminated because the MTD has been exceeded. For future trials, we intend to investigate the effects of 2 × 12.5 grams STS on infarct size in patients with STEMI (NCT02899364). This intended dose was chosen based on experimental data on the effects of H_2_S in the setting of ischemia-reperfusion injury, clinical data on STS, the elimination time of STS, logistical reasons, and the ongoing pathophysiological process of reperfusion injury [30]. In this pilot study, we aimed to evaluate also a higher dose cohort, as safety margin, and therefore defined our dosing endpoint fixed at 15 grams.

### 2.5. Statistical Analysis

Continuous data are reported as mean and standard deviation (SD) or median and interquartile range (IQR) depending on data distribution. Discrete variables were presented as frequencies and percentages. For outcome, DLT was expressed as categorical data and data on nausea/vomiting as ordinal data. SBP was assessed as a continuous variable and was compared within dose groups between consecutive time points and between dose cohorts on the same time points with linear mixed model analysis for the first dose and second dose separately. Additionally, the lowest, middle, and highest two dose cohorts were combined, and paired *t*-tests were performed to assess differences between two consecutive time points within these combined dose cohorts. In accordance with Benjamin et al., a two-tailed *P* value of <0.005 was considered statistically significant [31]. A *P* value between 0.05 and 0.005 was considered suggestive. STATA statistical software, release 15 (College Station, TX : StataCorp LLC), and SPSS statistics for Windows, version 23.0 (IBM Corp. Released 2015. Armonk, NY), were used for statistical analysis. Graphs were composed with GraphPad Prism version 7.0 for Windows (GraphPad Software, La Jolla California, USA).

## 3. Results

Baseline characteristics of the study population are presented in Table 1. A total of 18 patients were included. Of these, sixteen patients received two dosages 6 hours apart. Two patients received only 1 dosage of STS (STS 2.5 and 15 grams) because of early discharge from CCU. All patients had at least one troponin *T* value above the 99^th^ percentile upper reference limit. Fourteen patients were diagnosed with non-ST-segment elevation myocardial infarction (NSTEMI), two patients with unstable angina, and one patient had an aborted STEMI. 5 patients were classified with 0-vessel disease, and treatment was conservative: 4 patients presented with NSTEMI, due to transient thrombus (*n* = 1), microvascular disease (*n* = 1), spontaneous coronary artery dissection without any plaques or stenoses (*n* = 1), and the fourth patient had a history of STEMI without any new stenosis. In one patient, the diagnosis ACS was rejected, and troponin *T* rise was attributed to hypertension. From the remaining 13 patients, 10 patients (56%) underwent PCI. The other three patients had (prior) coronary artery disease without options for intervention. No patient underwent CABG. Before coronary angiography, 89% of the participants were treated with beta-blockers and 83% with ACE inhibitors or angiotensin II receptor blockers (Table 1). All patients received at least one additional antihypertensive drug (either beta-blocker or ACE inhibitor/angiotensin II receptor blocker or long-acting nitrate/nitroglycerin iv), next to the vasodilator radialis cocktail.

### 3.1. Primary and Secondary Outcomes

Primary and secondary outcomes are shown in Table 2. None of the patients reached primary endpoint, nor experienced a serious adverse event. No anaphylactic or infusion reactions occurred. Mild and transient nausea was reported in one patient. Two other patients reported moderate nausea, which already existed before administration of study medication. Metoclopramide was not prescribed in any patient.

### 3.2. Blood Pressure Measurements

**Figure 1:**
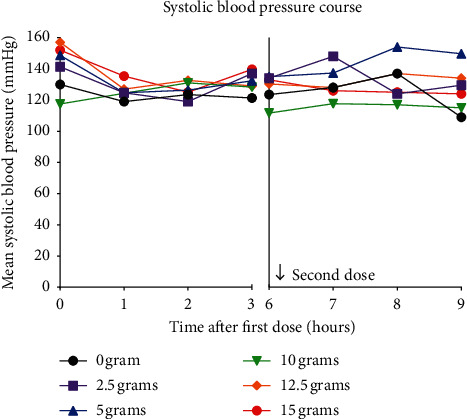
Systolic blood pressure course.

**Figure 2:**
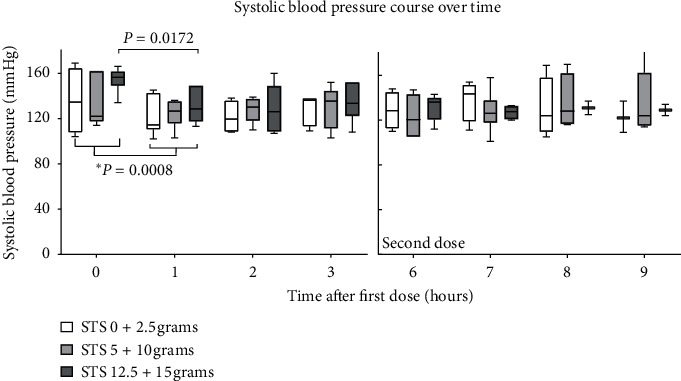
Systolic blood pressure course for combined dose cohorts.

Systolic blood pressure course at the predefined time points after the first and second dose for all dose cohorts is depicted in Figure 1. SBPs at the predefined time points were never lower than 101 mmHg (*t* = 7). Linear mixed modeling was performed for the first and second dose separately. No significant differences were observed between the different dose cohorts and over time (first dose: *P* = 0.844 over dose, *P* = 0.069 over time and second dose: *P* = 0.537 over dose, *P* = 0.478 over time). Additionally, paired *t*-tests were performed. A significant, but clinically well-tolerated, change between all SBPs before administration of study medication (*t* = 0, mean (SD), 142.5 (22.8) mmHg) and 1 hour after the first dose STS and verapamil/nitroglycerin cocktail (*t* = 1, 125.8 (14.7) mmHg) was observed (mean difference −16.8 mmHg; 95% confidence interval (CI) (−8.1 to −25.5); *P* = 0.0008; Figure 2). Subsequently, dose cohorts were combined (0 + 2.5 gr; 5 + 10 gr; and 12.5 + 15 gr), and paired *t*-tests were performed on blood pressures within those groups. Only for the highest combined dose cohort (12.5 + 15 gr), a suggestive decrease in SBP was observed from *t* = 0 to *t* = 1, (154.5 (10.9) vs. 131.2 (15.0) mmHg, mean difference −23.3; 95% CI (−6.2 to −40.4); *P* = 0.0172; Figure 2). No significant changes in SBP were observed after administration of the second dose STS, during which no concomitant vasodilatory medication was administered.

### 3.3. Adverse Events

In general, study medication was tolerated well. However, a few adverse events were observed besides our predefined endpoints. One patient experienced a vagal reaction due to wire manipulation during PCI (STS 2.5 gr), directly recovering after atropine administration. Two patients experienced a brief period of hypotension (SBP <90 mmHg), both lasting shorter than 25 minutes. The first patient experienced hypotension a few minutes after completion of study medication (STS 5 gr) and directly after stent placement in the left anterior descending artery, recovering after resuscitation with 1L NaCl 0.9%. The second patient experienced hypotension together with mild nausea right after a change of posture (from supine to sitting position), 15 minutes after administration of the second dose (STS 12.5 gr), with spontaneous recovery.

Both patients receiving only one dose did not experience an adverse event after administration of STS. Furthermore, their SBP 1 hour after administration of the first dose did not drop below 145 mmHg.

## 4. Discussion

This is the first study in which the H_2_S donor STS was administered in patients presenting with an ACS. We demonstrated that STS is well tolerated and appears to be safe in combination with concomitant administration of vasodilatory and blood pressure lowering drugs in ACS patients undergoing CAG via the transradial approach. Specifically, no clinically relevant changes in systolic blood pressure, dose-limiting toxicity or infusion reactions occur and side effects (nausea and hypotension) are mild and transient.

These observations are in line with previous experiences of STS use in human beings for other indications. In a study by Matthews et al., in which the feasibility, safety, and efficacy of STS in the progression of vascular calcification in hemodialysis patients was evaluated, escalating doses of STS (12.5 gr, 18.75 gr, and 25 gr) were administered during 30 minutes after each dialysis session [32]. STS infusions at the dose of 25 gr/treatment were associated with nausea and vomiting in all 22 patients despite antiemetic therapy. Final maintenance doses of 12.5 gr/treatment in 16 patients and 18.75 gr/treatment in 6 patients were well tolerated, and the authors concluded a dose of 12.5–18.75 gr to be feasible and safe, which is in line with our study. Also, a recent retrospective analysis of 24 calciphylaxis patients reported dose-limiting toxicity, mainly due to nausea and vomiting in two out of eighteen patients on 25 grams thrice weekly [33]. A dose lowering to 12.5 grams was well tolerated. However, these results are based on a retrospective analysis, and large prospective studies on optimal dosage and safety in calciphylaxis patients are lacking. For ototoxicity, two randomized trials have been carried out, in which doses of 16 gr/m2 and 20 gr/m2 were administered in 15 minutes [25, 26]. A total of 116 children, <18 years old, were treated with STS. In the first trial, one patient withdrew due to emesis and rigors. In the second trial, one patient developed severe metabolic acidosis and another patient suffered from severe nausea and vomiting. Most children tolerated doses of 20 gr/m2 (34 grams for an average adult body surface area of 1.7 m^2^) well, which is slightly higher than the maximum dose we investigated. However, extrapolation and application of these results to our study population should be performed with caution, since the other trials included severely ill children on chemotherapy with possibly different pharmacokinetic characteristics. However, also a dose-escalation pilot study in 29 patients (2–68 years old) for the prevention of ototoxicity from carboplatin treatment showed high doses (20 gr/m2; 34 grams for an average adult body surface area of 1.7 m^2^) to be well tolerated, albeit when antiemetic premedication was administered [34]. In this study, also low doses of 4 and 8 grams were already associated with nausea and vomiting. However, exact incidences were not reported.

New onset nausea was observed in our study in 5.6%. This is considerably lower than the ranges of nausea and vomiting reported in calciphylaxis patients. Two recent systematic reviews including over 700 patients treated with STS for calciphylaxis reported nausea and vomiting in 17–30% of the patients [27, 35]. The higher dosages used in calciphylaxis patients (25 gr thrice weekly) might be an explanation for this observed difference. Furthermore, hemodynamic effects of hemodialysis and metabolic imbalances within dialysis patients might be an additional explanation.

Most (case) studies do not report on blood pressure values after STS administration; however, mild hypotension is a known side effect, reported in 3.1% of patients in an analysis of multiple case reports from calciphylaxis patients (*n* = 64) [27]. In contrast, in a pilot study of 22 patients with end-stage renal disease, STS administration up to 25 grams was not associated with significant changes in blood pressure [32]. Effects of STS on blood pressure in experimental setting have been proven [36], but in human literature remain controversial. However, in human literature, when hypotension was reported, it was mild and manageable [37, 38]. In our study, a significant decrease in SBP was observed 1 hour after the first dose, especially in the higher dose cohort. However, blood pressure before study medication was already higher in the highest dose cohort. Therefore, we might be looking at a regression to the mean phenomenon. Moreover, after the second dose, no blood pressure decrease was observed, suggesting that the decrease in blood pressure after the first dose and coronary angiography might also be due to relieve of stress or the effect of concomitant administration of vasodilating drugs standard for the transradial procedure. However, this blood pressure decrease was not considered clinically relevant as it was mild, transient, and recovered spontaneously, except for one patient, which received limited fluid resuscitation. As reported earlier, none of our patients showed signs of cardiogenic shock, and no inotropic medication was needed.

Metabolic acidosis has been reported to occur in 7–30% of patients with calciphylaxis. We did not observe clinical signs of metabolic acidosis. Higher dosages of STS used for calciphylaxis, precarious acid/base balances, and high comorbidities in patients with end-stage renal disease might account for this observed difference. At last, in our study, we did not observe any anaphylactic reactions, nor has this been seen in previous studies in other patients' populations.

The intention of this study was to assess safety and tolerability of STS in the setting of ACS. Even though 5 patients did not present with (new) significant coronary artery stenosis (0-VD) and 8 patients did not undergo revascularization, all patients were concomitantly treated with vasodilators during transradial CAG and at least one blood pressure lowering drug. We demonstrated that in this setting of suspected ACS, STS administration appeared safe, and we could reasonably rule out procedure-related adverse events, especially severe hypotension. In SAFE-ACS, efficacy was not evaluated, and sample size was too small to draw conclusions on surrogate efficacy parameters such as CK levels. Considering the mechanism of action of STS, in the future GIPS-IV trial, the effect on myocardial infarct size, determined by magnetic resonance imaging, as surrogate endpoint for long-term outcomes (heart failure and survival) will be evaluated [39].

Limitations of our study are comparable to most pilot studies and comprise small sample size and relatively short follow-up. An additional limitation may be the inclusion of clinically stable ACS patients with mostly small infarctions. Therefore, conclusions on safety of STS treatment in other patients with ACS should be drawn with caution. However, the lack of clinically relevant (serious) adverse events suggests that this medication can be used relatively safe in a large group of patients with an ACS. Strengths of this study include the escalating dosage method, in which also higher dosages were evaluated than the desired one for future trials. Moreover, our patients were closely monitored for objectively chosen endpoints.

## 5. Conclusion

In conclusion, dosages of STS up to 2 × 15 grams, 6 hours apart, were well tolerated, and administration of STS appeared to be safe in patients with ACS treated with concomitant vasodilators and blood pressure lowering drugs. Our findings and encouraging preclinical data provide the basis for further clinical assessment on safety and efficacy of STS administration in patients with acute coronary syndromes.

## Figures and Tables

**Table 1:** Baseline characteristics.

Characteristics	Total (*n* = 18)
Age, mean ± SD (years)	62.2 ± 10.0
Male sex, *n* (%)	12 (66.7)
Body mass index, median (IQR) (kg/m^2^)	25.6 (23.3–27.4)
Ethnicity, *n* (%)	
Caucasian	17 (94.4)
African American	1 (5.6)
Cardiovascular-related history, *n* (%)	
Hypertension	12 (66.7)
Hypercholesterolemia	9 (50.0)
Smoking	7 (38.9)
Diabetes mellitus	4 (22.2)
Previous PCI	10 (55.6)
Previous CABG	1 (5.6)
Previous angina pectoris	7 (38.9)
Previous myocardial infarction	9 (50.0)
Stroke	2 (11.1)
Peripheral artery disease	1 (5.6)
Antihypertensive medications before PCI^a^, *n* (%)	
Beta-blocker	16 (89)
ACE inhibitor or angiotensin II receptor blocker	15 (83)
Calcium channel blocker	3 (17)
Diuretics	3 (17)
Long-acting nitrate or intravenous nitroglycerin	9 (50)
Blood pressure, mean ± SD (mmHg)	
Systolic	142 ± 22.8
Diastolic	82 ± 13.7
Heart rate, mean ± SD (beats/min)	70 ± 11.8
Infarct-related artery, *n* (%)	
Left main	0
Left circumflex coronary artery	2 (11.1)
Left anterior descending coronary artery	5 (27.8)
Right coronary artery	5 (27.8)
No clear culprit defined	6 (33.3)
Number of vessels affected, *n* (%)^b^	
0	5 (27.8)
1	7 (38.9)
2	6 (33.3)
Lab values at admission, median (IQR)	
High sensitive troponin *T* (ng/L)	23 (15–30)
CK (U/L)	95 (62–140)
CK-MB (U/L)	12 (10–17)
NT-proBNP (ng/L)	132 (53–262)
Creatinine (*μ*mol/L)	72 (66–84)

CK, creatine kinase; CK-MB, creatine kinase-myocardial band; IQR: interquartile range; NT-proBNP, N-terminal probrain natriuretic peptide; SD, standard deviation. ^a^Defined as antihypertensive drug use before admission or at least 1 dose <24 hours before arrival at the catheterization laboratory. ^b^≥70% reduction in the internal diameter of the right coronary artery, left anterior descending, or left circumflex coronary artery or ≥50% reduction in the internal diameter of the left main coronary artery.

**Table 2:** Outcome parameters.

Outcome	STS dose (grams)					
	0	2.5	5	10	12.5	15
Primary endpoint						
Dose-limiting toxicity	0	0	0	0	0	0

Secondary endpoints						
Anaphylaxis	0	0	0	0	0	0
Vomiting	0	0	0	0	0	0
Nausea						
Mild	0	0	0	0	1	0
Moderate	0	1^*∗*^	0	0	1^*∗*^	0
Severe	0	0	0	0	0	0

Peak values, median (IQR)						
Peak CK (U/L)	137 (87–227)					
Peak CK-MB (U/L)	23 (15–46)					
Peak high sensitive troponin *T* (ng/L)	80 (29–295)					

^*∗*^Moderate nausea complaints commenced before STS administration. CK, creatine kinase; CK-MB, creatine kinase-myocardial band; IQR, interquartile range; STS, sodium thiosulfate.

## Data Availability

The data used to support this study are made available from the corresponding author upon request.

## Supplementary Materials

Supplementary Materials Figure S1: Study design.

## References

[1] Yellon D. M., Hausenloy D. J. (2007). Myocardial reperfusion injury. *New England Journal of Medicine*.

[2] Heusch G., Libby P., Gersh B. (2014). Cardiovascular remodelling in coronary artery disease and heart failure. *The Lancet*.

[3] Davidson S. M., Ferdinandy P., Andreadou I. (2019). Multitarget strategies to reduce myocardial ischemia/reperfusion injury. *Journal of the American College of Cardiology*.

[4] Hausenloy D. J., Botker H. E., Engstrom T (2017). Targeting reperfusion injury in patients with ST-segment elevation myocardial infarction: trials and tribulations. *European Heart Journal*.

[5] Polhemus D., Butler J., Calvert J., Lefer D. (2014). The cardioprotective actions of hydrogen sulfide in acute myocardial infarction and heart failure. *Scientifica*.

[6] Elrod J. W., Calvert J. W., Morrison J. (2007). Hydrogen sulfide attenuates myocardial ischemia-reperfusion injury by preservation of mitochondrial function. *Proceedings of the National Academy of Sciences*.

[7] Snijder P. M., de Boer R. A., Bos E. M. (2013). Gaseous hydrogen sulfide protects against myocardial ischemia-reperfusion injury in mice partially independent from hypometabolism. *PLoS One*.

[8] Sodha N. R., Clements R. T., Feng J. (2009). Hydrogen sulfide therapy attenuates the inflammatory response in a porcine model of myocardial ischemia/reperfusion injury. *The Journal of Thoracic and Cardiovascular Surgery*.

[9] Calvert J. W., Jha S., Gundewar S. (2009). Hydrogen sulfide mediates cardioprotection through nrf2 signaling. *Circulation Research*.

[10] Sun W.-H., Liu F., Chen Y., Zhu Y.-C. (2012). Hydrogen sulfide decreases the levels of ROS by inhibiting mitochondrial complex IV and increasing SOD activities in cardiomyocytes under ischemia/reperfusion. *Biochemical and Biophysical Research Communications*.

[11] Chen S.-L., Yang C.-T., Yang Z.-L. (2010). Hydrogen sulphide protects H9c2 cells against chemical hypoxia-induced injury. *Clinical and Experimental Pharmacology and Physiology*.

[12] Alvert J. W., Elston M., Nicholson C. K. (2010). Genetic and pharmacologic hydrogen sulfide therapy attenuates ischemia-induced heart failure in mice. *Circulation*.

[13] Qipshidze N., Metreveli N., Mishra P. K., Lominadze D., Tyagi S. C. (2012). Hydrogen sulfide mitigates cardiac remodeling during myocardial infarction via improvement of angiogenesis. *International Journal of Biological Sciences*.

[14] Simon F., Giudici R., Duy C. N. (2008). Hemodynamic and metabolic effects of hydrogen sulfide during porcine ischemia/reperfusion injury. *Shock*.

[15] Li H., Sun W., Li L., Xu C. (2016). Exogenous H_2_S contributes to recovery of ischemic post-conditioning-induced cardio protection in the aging rat and cardiomyocytes and the related mechanism. *Journal of Molecular and Cellular Cardiology*.

[16] Olson K. R., Whitfield N. L. (2010). Hydrogen sulfide and oxygen sensing in the cardiovascular system. *Antioxidants & Redox Signaling*.

[17] Kruithof P. D., Lunev S., Aguilar Lozano S. P. (2020). Unraveling the role of thiosulfate sulfurtransferase in metabolic diseases. *Biochimica et Biophysica Acta (BBA)-Molecular Basis of Disease*.

[18] Kannan S., Boovarahan S. R., Rengaraju J. (2019). Preconditioning the rat heart with sodium thiosulfate preserved the mitochondria in response to ischemia-reperfusion injury. *Journal of Bioenergetics and Biomembranes*.

[19] Ravindran S., Kurian G. A. (2019). Preconditioning the rat heart with sodium thiosulfate preserved the mitochondria in response to ischemia-reperfusion injury. *Journal of Bioenergetics and Biomembranes*.

[20] Ravindran S., Kurian G. A., Ramachandran K., Kurian G. A. (2018). Effect of sodium thiosulfate postconditioning on ischemia-reperfusion injury induced mitochondrial dysfunction in rat heart. *Journal of Cardiovascular Translational Research*.

[21] Ravindran S., Ramachandran K., Kurian G. A. (2018). Sodium thiosulfate mediated cardioprotection against myocardial ischemia-reperfusion injury is defunct in rat heart with co-morbidity of vascular calcification. *Biochimie*.

[22] Ravindran S., Jahir Hussain S., Boovarahan S. R. (2017). Sodium thiosulfate post-conditioning protects rat hearts against ischemia reperfusion injury via reduction of apoptosis and oxidative stress. *Chemico-Biological Interactions*.

[23] Chen K. K., Rose C. (1952). Nitrite and thiosulfate therapy in cyanide poisoning. *Journal of the American Medical Association*.

[24] Kyriakopoulos G., Kontogianni K. (1190). Sodium thiosulfate treatment of tumoral calcinosis in patients with end-stage renal disease. *Renal Failure*.

[25] Brock P. R., Maibach R., Childs M. (2018). Sodium thiosulfate for protection from cisplatin-induced hearing loss. *New England Journal of Medicine*.

[26] Freyer D. R., Chen L., Krailo M. D. (2017). Effects of sodium thiosulfate versus observation on development of cisplatin-induced hearing loss in children with cancer (ACCL0431): a multicentre, randomised, controlled, open-label, phase 3 trial. *The Lancet Oncology*.

[27] Peng T., Zhuo L., Wang Y. (2018). Systematic review of sodium thiosulfate in treating calciphylaxis in chronic kidney disease patients. *Nephrology*.

[28] Ponikowski P., Voors A. A., Anker S. D. (2016). 2016 ESC Guidelines for the diagnosis and treatment of acute and chronic heart failure. *European Heart Journal*.

[29] Simons F. E. R., Ebisawa M., Sanchez-Borges M. (2015). 2015 update of the evidence base: World Allergy Organization anaphylaxis guidelines. *World Allergy Organization Journal*.

[30] Vinten-Johansen J. (2004). Involvement of neutrophils in the pathogenesis of lethal myocardial reperfusion injury. *Cardiovascular Research*.

[31] Benjamin D. J., Berger J. O., Johannesson M. (2018). Redefine statistical significance. *Nature Human Behaviour*.

[32] Mathews S. J., De Las Fuentes L., Podaralla P. (2011). Effects of sodium thiosulfate on vascular calcification in end-stage renal disease: a pilot study of feasibility, safety and efficacy. *American Journal of Nephrology*.

[33] Harris C., Kiaii M., Lau W., Farah M. (2018). Multi-intervention management of calcific uremic arteriolopathy in 24 patients. *Clinical Kidney Journal*.

[34] Neuwelt E. A., Brummett R. E., Doolittle N. D (1998). First evidence of otoprotection against carboplatin-induced hearing loss with a two-compartment system in patients with central nervous system malignancy using sodium thiosulfate. *The Journal of Pharmacology and Experimental Therapeutics*.

[35] Udomkarnjananun S., Kongnatthasate K., Praditpornsilpa K., Eiam-Ong S., Jaber B. L., Susantitaphong P. (2019). Treatment of calciphylaxis in ckd: a systematic review and meta-analysis. *Kidney International Reports*.

[36] Nguyen I. T. N., Klooster A., Minnion M. (2020). Sodium thiosulfate improves renal function and oxygenation in L-NNA-induced hypertension in rats. *Kidney International*.

[37] Thomas J. E., McGinnis G. (2002). Safety of intraventricular sodium nitroprusside and thiosulfate for the treatment of cerebral vasospasm in the intensive care unit setting. *Stroke*.

[38] Malbos S., Ureña-Torres P., Bardin T., Ea H.-K. (2016). Sodium thiosulfate is effective in calcific uremic arteriolopathy complicating chronic hemodialysis. *Joint Bone Spine*.

[39] Ibanez B., Aletras A. H., Arai A. E. (2019). Cardiac MRI endpoints in myocardial infarction experimental and clinical trials. *Journal of the American College of Cardiology*.

