# Supplementary figures and images for: Safety and Tolerability of Sodium Thiosulfate in Patients with an Acute Coronary Syndrome Undergoing Coronary Angiography: A Dose-Escalation Safety Pilot Study (SAFE-ACS)
Source: J Interv Cardiol. 2020 Sep 24;2020:6014915. doi: 10.1155/2020/6014915 (PMC7532357; doi:10.1155/2020/6014915)

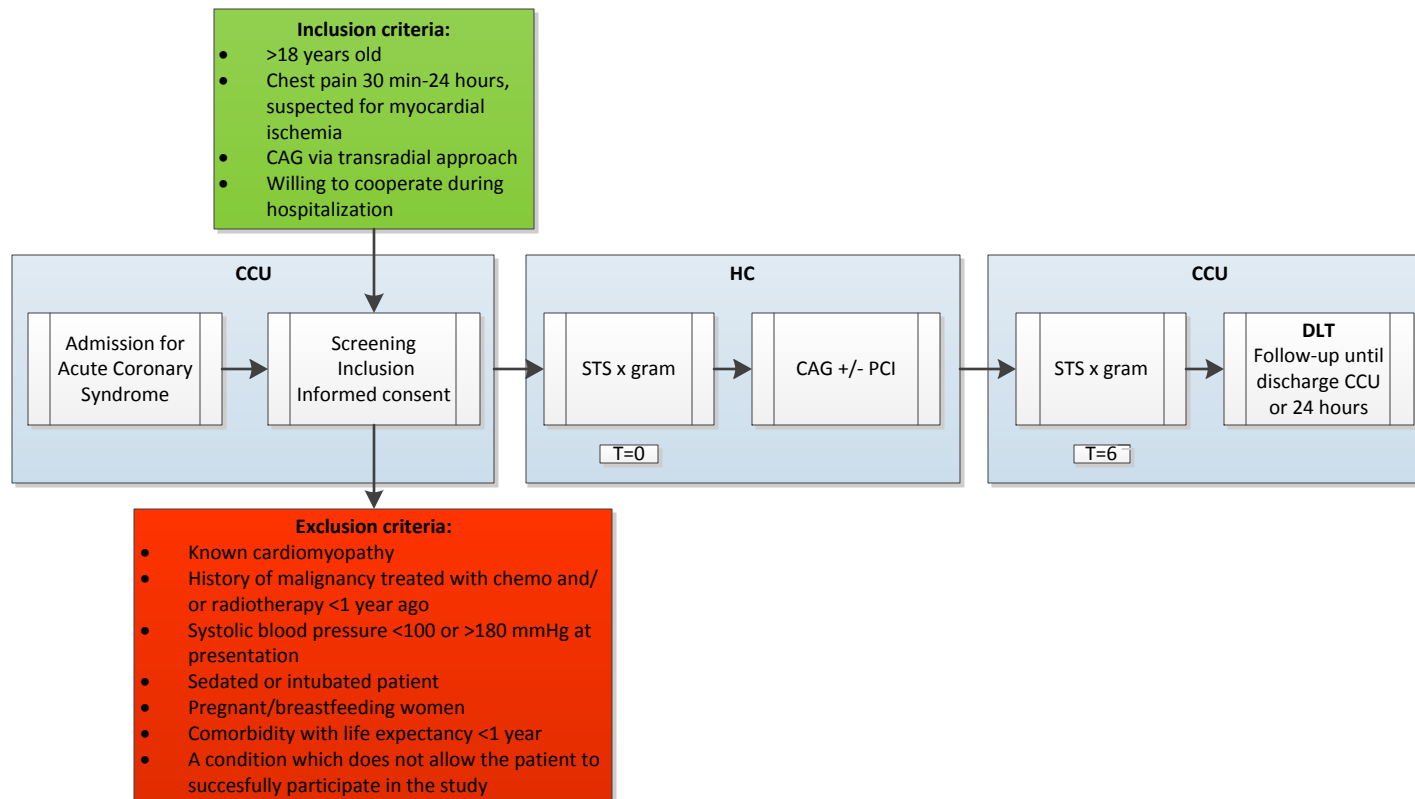

Supplement: Supplementary Materials — Figure S1: Study design. [file 6014915.f1.pdf]
